# Rhabdomyolysis and Acute Kidney Injury due to Severe Heat Stroke

**DOI:** 10.1155/2011/951719

**Published:** 2011-09-27

**Authors:** Máximo H. Trujillo, Carlos Fragachán G.

**Affiliations:** ^1^Critical Care Department, ICU, Instituto Médico La Floresta (IMLF), Caracas 1060, Venezuela; ^2^Critical Care Department, University Hospital of Caracas, Caracas 1050, Venezuela

## Abstract

We present a case of heat stroke (HS) and acute kidney injury (AKI) due to severe rhabdomyolysis in a 14-year-old previously healthy female patient. When she was practicing strenuous exercise she suffered acute seizures and high fever. These symptoms were followed by coma and multiple organ failure (MOF), which included AKI, encephalopathy, fulminant hepatic failure (FHF), and disseminated intravascular coagulation (DIC). The patient was managed in the ICU with renal replacement therapy, ventilatory support, and other vital supporting measures. After three weeks of ICU treatment she made a full recovery.

## 1. Introduction

Rhabdomyolysis is a syndrome characterized by the leakage of muscle-cell contents into the circulation. Strenuous exercise and HS are the most common causes of rhabdomyolysis. The severity of illness ranges from asymptomatic elevations of the serum levels of muscle enzymes to life-threatening cases associated with extreme elevations of creatine kinase (CK) and liver enzymes (ALT, AST), electrolyte imbalances, and AKI. There are two forms of rhabdomyolysis: traumatic, often resulting from crush injuries and extensive trauma sustained in natural disasters and wars and nontraumatic, which include, among other causes, excessive physical exertion, HS, exogenous toxins, usage of illicit drugs, malignant hyperthermia, alcohol, and prescription medications. The risk of AKI in rhabdomyolisis is usually low when CK levels on admission are less than 20000 U/L. Nevertheless, AKI is still at risk with values of CK as low as 5000 U/L when other coexisting conditions occurs such as sepsis, severe dehydration, and acidosis [1–4]. In addition to rhabdomyolisis, all three risk factors may coexist in severe HS. Usually the cause of rhabdomyolysis is evident from the patient's history or from circumstances immediately preceding the disorder, and only occasionally the precipitant cause will not be obvious.

The classic presentation of rhabdomyolysis includes myalgias, limb weakness, and pigmenturia due to myoglobinuria without hematuria. AKI is a potential complication of severe rhabdomyolysis independently of its etiology, which accounts for 8–15% of all cases of AKI in the US. The mortality rate of HS can be as high as 70%, especially when treatment is delayed, and the presence of MOF [5–9]. Elevated serum creatinine and oliguria associated with rhabdomyolysis and coagulopathy in HS further increase the rate of AKI and mortality [8].

## 2. Report of the Case

On admission to the ICU her physical examination revealed a temperature of 38°C, blood pressure of 160/90 mmHg, pulse rate of 150/min, and respiratory rate of 35/min. Oxygen saturation was 90%. Except for dry mucous membranes, the rest of her physical examination was unremarkable. Soon after admission to the ICU she became unconscious, requiring tracheal intubation and ventilatory support. A chest X-ray and EKG were normal. A cerebral CT scan revealed mild cerebral edema in both hemispheres.

Laboratory testing revealed serum creatinine 2.4 mg/dL, blood urea nitrogen (BUN) 104 mg/dL, potassium 3 mEq/L, sodium 150 mEq/L, CPK level 24,385 U/L, serum myoglobin >3000 ng/mL, total bilirubin 3.12 mg/dL (d/i: 1.49/1.71), ALAT 382 U/L and AST of 282 U/L, and LDH of 1,376 U/L; serum ammonium 16 mmol/L (reference values 10–80 mmol/L); INR 2.73, and serum fibrinogen of 203 mg/dL. The urinalysis revealed reddish brown urine highly positive for myoglobin. The diagnosis of severe exertional HS and rhabdomyolysis with ensuing AKI was established. The patient received IV hydration, ventilatory support, sodium bicarbonate, corticosteroids, and enteral nutrition. During the next 3 days, her CPK level increased to 36,423 U/L, the serum myoglobin >3000 ng/mL, ALAT 9,942 U/L, AST 5,910 U/L, and LDH to 4,623 U/L. Serum ammonium 117 mmol/L, INR 4.7, and the total bilirubin 11.9 mg/dL (d/i: 9.6/2.3). These last laboratory findings and the onset of encephalopathy and coagulopathy in a patient with not known previous liver disease were consistent with the diagnosis of FHF.

The platelet count decreased to 18,000/mm^3^, the plasma fibrinogen to 104 mg/dL, and the fibrinogen split products were 20 *μ*g/mL (<10 *μ*g/mL), supporting the diagnosis of DIC without evidence of active bleeding. On the fourth day, the patient became oliguric (<20 mL/h) and the serum creatinine increased to 5.6 mg/dL with BUN of 118 mg/dL. The diagnosis of AKI was definitively established, and daily hemodialysis was started and maintained for 17 days. By the 5th hospitalization day she had a maximum APACHE II and SAPS scores of 37 and 78 points, respectively. By the 12th day in the ICU, the urine flow started a progressive increase, reaching a maximum of 5 L/24 h. This was accompanied by a decrease of serum creatinine to 2.3 mg/dL and BUN to 38 mg/dL. Hemodialysis was then discontinued. Simultaneously, serum levels of bilirubin, ammonium, ALAT, AST, CK, and myoglobin showed a progressive decrease to normal values. The patient was weaned from the ventilator and extubated on her 14th day of admission to the ICU. Her CNS involvement improved steadily, and complete recovery was attained by the 25th day of hospitalization. Further recovery was uneventful, and she was discharged home. After 3 weeks, she returned to school and normal activities.

## 3. Discussion

Two forms of HS have been described: the classic or nonexertional HS, which occurs frequently during environmental heat waves, and exertional HS, which usually occurs in healthy, young, and physically active people who engage in strenuous exercise. Renal dysfunction is a well-documented feature in HS that occurs in 30% of cases approximately. The etiology is multifactorial, and this includes direct thermal injury, rhabdomyolysis, DIC, and renal hypoperfusion due to volume depletion and arterial hypotension [10–13]. AKI associated with myoglobinuria is the most serious complication of both traumatic and nontraumatic rhabdomyolysis and may be life threatening. AKI as a complication of rhabdomyolysis is quite common, representing about 7–10% of all cases of AKI in the United States [14], but the incidence of AKI in rhabdomyolysis may be as high as 50% [3, 4, 14]. 

In spite of early volume replacement and correction of transient arterial hypotension, AKI was an early feature in the present case, suggesting that rhabdomyolysis played a central role in the pathophysiology of this complication. Massive rhabdomyolysis may follow strenuous physical exertion, particularly when associated with other risk factors such as extremely hot and humid conditions, poor physical fitness, and hypokalemia [5, 15]. Apart from severe dehydration and fluid sequestration in injured muscles, AKI in the context of rhabdomyolysis results from a combination of factors. These include direct tubular toxicity, ischemia caused by circulating vasoconstrictors, activation of the sympathetic nervous system, antidiuretic hormone, and the renin-angiotensin system [16]. Myoglobin itself seems to have no marked nephrotoxic effect on the tubules unless the urine is acid. In which case it is reasonable to consider alkalinization of urine as a therapeutic measure, especially in patients with metabolic acidosis [14, 16]. When serum myoglobin levels reach 100 mg/dL, the urine becomes reddish-brown in color. In establishing a differential diagnosis of myoglobinuria, other causes of pigmenturia should be considered (Table 1). 

One of the first steps in managing patients with HS in addition to rapid body cooling is prompt IV repletion of fluids. However, once renal damage supervenes, as occurred with our patient, renal replacement therapy is required to control hyperkalemia, fluid overload, and uremia. The outcome of rhabdomyolysis is usually good provided there is no renal failure. Mortality data vary widely according to severity and coexisting conditions. In cases of rhabdomyolysis secondary to limb ischemia (crush injury) mortality can be as high as 32% [17], and among patients hospitalized in intensive care units mortality has been reported to be as high as 59% when AKI is present and 22% when it is not [18]. In contrast, long-term survival among patients with AKI due to rhabdomyolysis is close to 80%, and the majority of them usually recover full renal function [19]. 

Liver dysfunction and increased serum levels of liver enzymes are commonly observed in HS, whereas FHF is a rare event FHF, defined as ‘‘the onset of encephalopathy and coagulopathy in patients with no known previous liver disease,” is very uncommon in HS, and only three reports of such cases have been previously published. Two of them recovered completely with conservative ICU treatment [20, 21]. Our patient also made a full recovery from FHF, suggesting that both liver injury and AKI in exertional HS may be reversible. 

The severity and complications of exertional HS correlate with the temperature duration curve, suggesting that a rapid reduction in the core temperature by any cooling method (immersion in iced water, iced peritoneal, or gastric lavage) as early as possible should have a positive effect on prognosis. In fact, this single measure seems to be the most effective way of preventing MOF in HS patients. Unfortunately, the ‘‘golden hour” for our patient had probably passed by the time she arrived at the ICU [22]. We believe that had she received rapid cooling measures immediately on admission to the suburban hospital, the extent of organ damage may have been limited.

Although rapid reduction of body temperature and repletion of body fluids remain the vital first priority in the treatment of HS, this together with vigorous intensive care management and vital supporting measures may result in patient survival and complete recovery. Nevertheless, to date little is known about the prognosis of patients with HS, and more data are needed to determine the most appropriate and effective management strategies for these cases.

## Figures and Tables

**Table 1:** Differential diagnosis of pigmenturia.

	Hematuria	Hemoglobinuria	Myoglobinuria	Bile pigments	Porphyria	Alkaptonuria

Pigment	RBC*	Hemoglobin	Myoglobin	Bilirubin/Urobilin	Porphobilinogen	Homogentisinic acid
Urine color	Red	Pink	Red to brown	Brown	Turns brown, red, purple or black on standing at sunlight. Fluoresces with UV light	Turns dark in alkaline solutions. Darkens on standing at sunlight.
Urine Sedi-ment***	RBC and RBC casts	Normal**	Normal	Normal	Normal	Normal
Supernatant urine color	Yellow	Red to brown	Red to brown	Brown	Red. (Watson-Schwartz test positive)	Normal (Ferric chloride test positive)
Urine dipstick test****	1 to 4 +	1 to 4 +	1 to 4 +	Normal	Normal	Normal
Serum	Normal	Pink (low haptoglobin)	Normal (increase ofmyoglobin,creatinine kinase, and liverenzymes inrhabdomyolysis)	Icteric	Normal	Normal
Muscle symptoms	No	No	Myalgias	No	Abdominal cramps.	No (ochronosis and arthritis)

*RBC red blood cells.

**Normal refers to white or yellow in color.

***The sediment and supernatant urine examined after centrifugation.

****Semiquantitative test (orthotolidine or peroxidase) detects heme peroxidase activity in RBC, hemoglobin or myoglobin with reported sensitivity of 91–100%. (1 + = 5–10 RBC/*μ*L, 4+ = approx. 250 RBC/*μ*L).
